# 3-Mercaptopyruvate sulfurtransferase produces potential redox regulators cysteine- and glutathione-persulfide (Cys-SSH and GSSH) together with signaling molecules H_2_S_2_, H_2_S_3_ and H_2_S

**DOI:** 10.1038/s41598-017-11004-7

**Published:** 2017-09-05

**Authors:** Yuka Kimura, Shin Koike, Norihiro Shibuya, David Lefer, Yuki Ogasawara, Hideo Kimura

**Affiliations:** 1 National Institute of Neuroscience, National Center of Neurology and Psychiatry, 4-1-1 Ogawahigashi, Kodaira, Tokyo 187-8502 Japan; 2 Department of Analytical Biochemistry, Meiji Pharmaceutical University, 2-552-1 Noshio, Kiyose, Tokyo 204-8588 Japan; 3 Department of Pharmacology and Experimental Therapeutics and Cardiovascular Center of Excellence, LSU Health Science Center, New Orleans, LA 70112 USA

## Abstract

Cysteine-persulfide (Cys-SSH) is a cysteine whose sulfhydryl group is covalently bound to sulfur (sulfane sulfur). Cys-SSH and its glutathione (GSH) counterpart (GSSH) have been recognized as redox regulators, some of which were previously ascribed to cysteine and GSH. However, the production of Cys-SSH and GSSH is not well understood. Here, we show that 3-mercaptopyruvate sulfurtransferase (3MST) produces Cys-SSH and GSSH together with the potential signaling molecules hydrogen per- and tri-sulfide (H_2_S_2_ and H_2_S_3_). Cys-SSH and GSSH are produced in the brain of wild-type mice but not in those of 3MST-KO mice. The levels of total persulfurated species in the brain of 3MST-KO mice are less than 50% of that in the brain of wild-type mice. Purified recombinant 3MST and lysates of COS cells expressing 3MST showed that Cys-SSH and GSSH were produced in the presence of physiological concentrations of cysteine and glutathione, while those with longer sulfur chains, Cys-SS_n_H and GSS_n_H, were produced in the presence of lower than physiological concentrations of cysteine and glutathione. The present study provides new insights into the production and physiological roles of these persulfurated species as well as the therapeutic targets for diseases in which these molecules are involved.

## Introduction

Cysteine persulfide (Cys-SSH) together with its glutathione (GSH) counterpart (GSSH) have been proposed to be involved in redox homeostasis. Cys-SSH and GSSH have been reported to reduce cytochrome c more efficiently than cysteine or GSH^1^. The potent reducing activity and scavenging effect of GSSH was also demonstrated on papain^2^. The production of Cys-SSH from cystine by cystathionine γ–lyase (CSE) had initially been demonstrated by Cavallini *et al*.^3^, and cystathionine β–synthase (CBS) was recently proposed to have activity similar to CSE^4^. However, both CSE and CBS are localized in the cytoplasm where cysteine is the dominant form over cystine. Based on these observations it is controversial whether CSE and CBS produce Cys-SSH in cells, and the identification of the Cys-SSH-producing enzyme has been anticipated^5^.

Hydrogen per- and tri-sulfide (H_2_S_2_ and H_2_S_3_) have recently emerged as novel signaling molecules related to hydrogen sulfide (H_2_S), which regulates neuronal transmission, vascular tone, cytoprotection, anti-inflammation, and oxygen sensing^6–13^. Recently, we demonstrated that H_2_S_2_ and H_2_S_3_ as well as H_2_S are produced by 3-mercaptopyruvate sulfur transferase (3MST)^14^. H_2_S_2_ and H_2_S_3_ are also generated by the interaction of H_2_S with nitric oxide (NO)^15–18^. H_2_S_n_ (n ≥ 2) exert various physiological roles such as activating transient receptor potential ankyrin 1 (TRPA1) channels to induce Ca^2+^ influx in astrocytes and dorsal root ganglion neurons^18–22^. They facilitate the translocation of nuclear factor-like 2 (Nrf2) to the nucleus by modifying its binding partner kelch-like ECH-associated protein 1 (Keap1)^23^. They also activate protein kinase G1α to regulate vascular tone^24^ and regulate tumor suppressor phosphatase and tensin homolog (PTEN)^25^. Some of these activities were previously thought to be mediated by H_2_S produced by CBS and CSE as well as 3MST^26–30^.

Proteins such as super oxide dismutase 1 and growth hormone have persulfurated cysteine residues in their structures^31–34^. Parkin, an E3 ubiquitin ligase whose mutations are the most common cause of hereditary Parkinson’s disease (PD), is less persulfurated in the PD brain than in the normal brain^13, 35^. Hylin and Wood reported that the persulfurated cysteine residues of proteins can be produced from 3-mercaptopyruvate (3MP), a substrate of 3MST^36^.

The total persulfurated species in cells or tissues such as Cys-SSH, GSSH, Cys-SS_n_H, GSS_n_H, H_2_S_n_ in addition to persulfurated cysteine residues of proteins, have been designated as bound sulfane sulfur^37–39^. The levels of bound sulfane sulfur were increased in cells expressing 3MST, while they were not increased in cells expressing defective 3MST mutants^26^. Recently, we demonstrated that 3MST produced H_2_S_2_ and H_2_S_3_^14^. Because H_2_S_n_ are greatly reactive, it is possible that H_2_S_n_ immediately react with intracellular cysteine, GSH, and cysteine residues of proteins to produce Cys-SSH, GSSH, and persulfurated cysteine residues^40^. Alternatively, 3MST may transfer sulfur from 3MP to cysteine and GSH to produce these persulfurated species.

The present study shows that 3MST produces Cys-SSH, GSSH, and persulfurated cysteine residues of proteins under physiological conditions together with H_2_S_n_ and H_2_S. It provides new insights into the production of reactive persulfurated species that mediate various cellular signaling processes, and identifies therapeutic targets in diseases where these molecules are involved.

## Results

### Production of bound sulfane sulfur by 3MST

We previously showed that cells expressing 3MST contain greater levels of bound sulfane sulfur than control cells or those expressing defective 3MSTs, which lost the ability to produce H_2_S and H_2_S_n_^14, 26^. In contrast, CBS, which produces H_2_S, did not increase the level of bound sulfane sulfur^26, 38, 39^. These observations suggest that 3MST but not that CBS produces persulfurated species.

**Figure 1:**
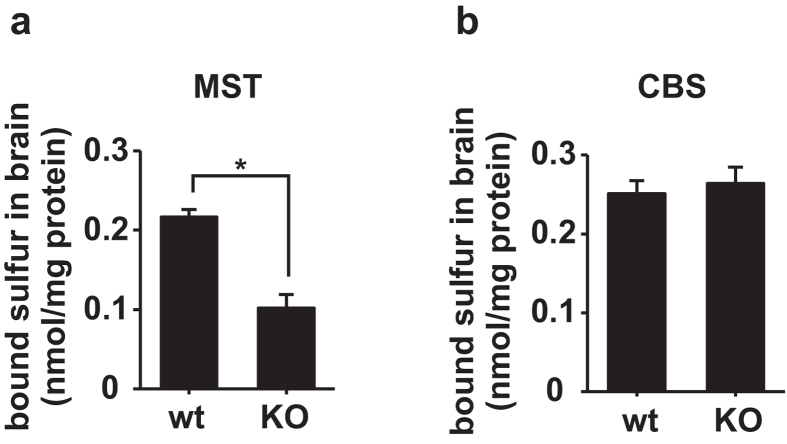
Levels of bound sulfane sulfur in mouse brains. (**a**) The bound sulfane sulfur level in the brains of 3MST-KO (KO) mice is significantly lower than that in the brains of wild type (wt) mice. *p < 0.05 by ANOVA (n = 3). (**b**) There is no significant difference in bound sulfane sulfur levels between CBS-KO (KO) and wild type (wt) mice (n = 4). All data expressed as mean + s.e.m.

In order to confirm these results in the brain, we compared the levels of bound sulfane sulfur in 3MST-KO mice to those in wild type mice^14^. The levels of bound sulfane sulfur in 3MST-KO brain were less than approximately 50% in wild-type brains (Fig. 1a). In contrast, bound sulfane sulfur in CBS-KO brains was not significantly different from that in wild-type brains (Fig. 1b)^41^. These observations confirm that 3MST produces bound sulfane sulfur, while CBS does not^26^. Because the expression of CSE in the brain is under the detectable threshold, contribution of this enzyme to the production of bound sulfane sulfur must be negligible^42, 43^.

### H_2_S_n_ react with cysteine and GSH to produce Cys-SS_n_H and GSS_n_H

**Figure 2:**
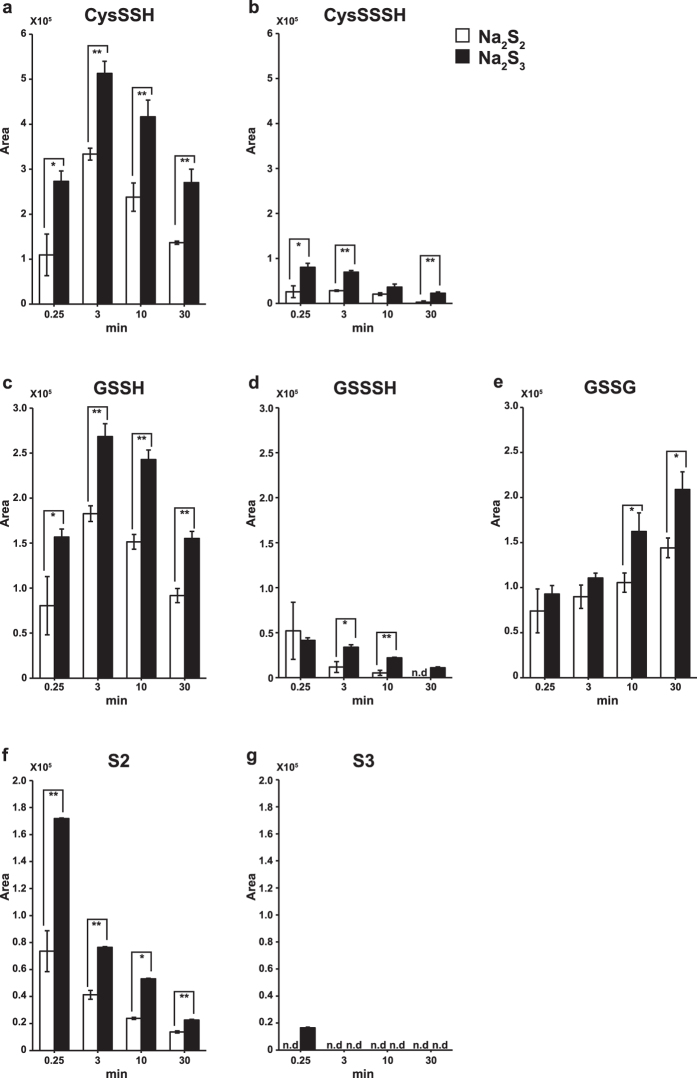
Chemical interaction of H_2_S_n_ with cysteine and GSH generates Cys-SS_n_H and GSS_n_H. (**a**–**g**) Cys-SSH (**a**), Cys-SSSH (**b**), GSSH (**c**), GSSSH (**d**), GSSG (**e**), H_2_S_2_ (**f**) and H_2_S_3_ (**g**) produced after 0.25, 3, 10 and 30 min after the application of Na_2_S_2_ (final concentration of 10 μM, open bar) or Na_2_S_3_ (filled bar) to the medium containing 100 μM cysteine and 1 mM GSH. **p < 0.01, *p < 0.05 (n = 3) by Student t-test. All data expressed as mean ± s.e.m. N. d.: Not detected.

We previously showed that cells expressing 3MST produce H_2_S_2_ and H_2_S_3_ from 3MP^14^. Because H_2_S_n_ are highly reactive, it is possible that intracellular cysteine and GSH immediately react with H_2_S_n_ to produce Cys-SSH and GSSH. We examined this possibility. Na_2_S_2_ or Na_2_S_3_ was added to make the final concentration of 10 μM to the medium containing 100 μM cysteine and 1 mM GSH, and the production of Cys-SS_n_H and GSS_n_H was examined. Cys-SSH, Cys-SSSH, GSSH, and GSSSH were produced 15 sec after the addition of Na_2_S_2_ or Na_2_S_3_ and their levels were increased until 3 min, and then declined thereafter (Fig. 2a–d). H_2_S_3_ was detected only 15 sec after the addition of Na_2_S_3_, and only H_2_S_2_ was detected thereafter (Fig. 2f and g). Na_2_S_3_ produces Cys-SSH, Cys-SSSH, GSSH, and GSSSH more efficiently than Na_2_S_2_, and H_2_S_3_ stays in the medium much more shortly than H_2_S_2_ due to its high reactivity (Fig. 2f and g). These observations suggest that H_2_S_n_ immediately react with cysteine and GSH to generate mainly Cys-SSH and GSSH in the presence of endogenous concentrations of cysteine and GSH. Although GSSG was produced, cystine and H_2_S were not detected in the reaction mixture (Fig. 2e).

### Cysteine and GSH are decreased when H_2_S_n_ are produced

**Figure 3:**
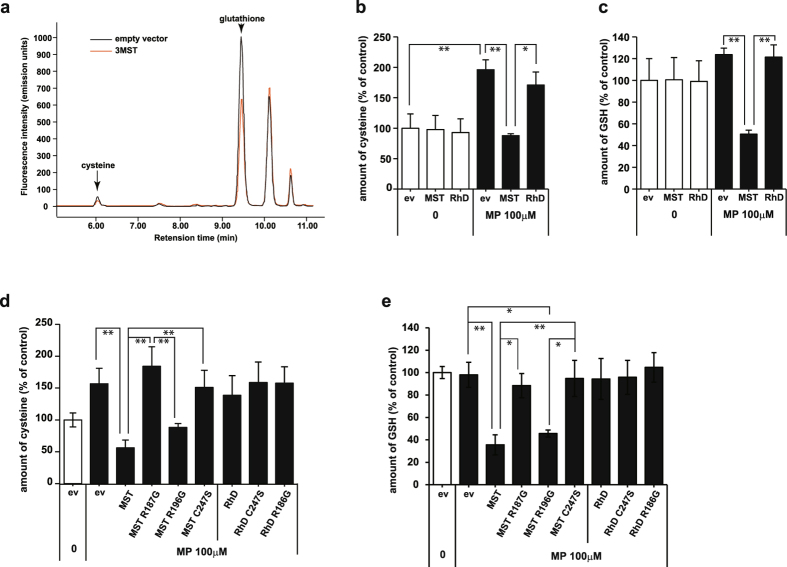
Cysteine and GSH levels were decreased in lysates of COS cells expressing 3MST in the presence of 3MP. (**a**) A representative HPLC chart of monobromobimane adducts of cell lysates. Cysteine and GSH were decreased in lysates of cells expressing 3MST compared with those transfected with an empty vector. Note that the reaction mixture of lysates contained approximately 1 μM cysteine and 10 μM GSH. (**b**,**c**) The levels of cysteine (**b**) and GSH (**c**) in cells expressing 3MST in the presence of 100 μM 3MP were significantly lower than those in control cells (n = 5). (**d**,**e**) Levels of cysteine (**d**) and GSH (**e**) in lysates of cells expressing various mutants of 3MST and rhodanese (Rhd) in the presence of 100 μM 3MP (n = 3). **p < 0.01, *p < 0.05 by ANOVA. All data expressed as mean ± s.e.m.

Because of the high reactivity of H_2_S_n_ with cysteine and GSH to produce Cys-SS_n_H and GSS_n_H (Fig. 2), it is possible that cysteine and GSH are consumed when H_2_S_n_ are produced^40^. We examined this possibility using lysates of COS cells expressing 3MST in the presence or absence of 3MP. The reaction mixture of lysates contained approximately 1 μM cysteine and 10 μM GSH. In the presence of 3MP, the levels of cysteine and GSH were greatly decreased in lysates of cells expressing 3MST compared with control cell lysates (Fig. 3a–c), suggesting that cysteine and GSH are consumed to produce Cys-SSH and GSSH. The levels of cysteine were greatly increased in control cell lysates in the presence of 3MP. This is probably due to endogenous cysteine aminotransferase, which converts 3MP to cysteine (Fig. 3b)^44^.

GSH levels were also dramatically decreased in the presence of 3MP in cell lysates expressing 3MST (Fig. 3c). Rhodanese, which is also sulfurtransferase with approximately 60% amino acid homology with 3MST, did not change the levels of cysteine nor GSH (Fig. 3b and c)^45^. These observations suggest that cysteine and GSH are consumed by reacting with H_2_S_n_ to produce Cys-SSH and GSSH.

In order to investigate whether the catalytic activity of 3MST is involved in decreasing the level of cysteine and GSH, the effect of 3MST defective mutants were examined. A catalytic site mutant 3MST C247S, which has lost the activity to produce H_2_S and H_2_S_n_, did not decrease cysteine levels (Fig. 3d)^14, 28, 45^. Another mutant R187G showed a similar result to C247S. R196G, which preserves 3MST activity to some extent, decreased the level of cysteine (Fig. 3d). Rhodanese and its defective mutants did not have an effect on the level of cysteine even in the presence of 3MP (Fig. 3d).

A similar result was also obtained for the levels of GSH. 3MST and R196G also decreased the GSH level in the presence of 3MP, while C247S and R187G did not (Fig. 3e). Rhodanese and its mutants did not have any effect on the level of GSH (Fig. 3e). These observations suggest that the catalytic activity of 3MST is required for the consumption of cysteine and GSH.

Although it is less efficient compared with 3MP as a substrate, 3MST also facilitates the production of H_2_S_n_ from H_2_S^14^. We examined the consumption of cysteine and GSH by 3MST in the presence of Na_2_S, the sodium salt of H_2_S. The levels of cysteine and GSH were slightly decreased in cells expressing 3MST in the presence of Na_2_S, but not in a statistically significant quantity (Supplementary Fig. S1a and b). This is probably due to the less efficient production of H_2_S_n_ from H_2_S compared to that from 3MP. It may also be due to the reducing activity of H_2_S, which immediately reduces Cys-SSH and GSSH back to cysteine and GSH upon production. No significant change in the levels of cysteine and GSH were observed in cells expressing rhodanese in the presence of H_2_S (Supplementary Fig. S1a and b).

### Production of Cys-SSH and GSSH by 3MST

**Figure 4:**
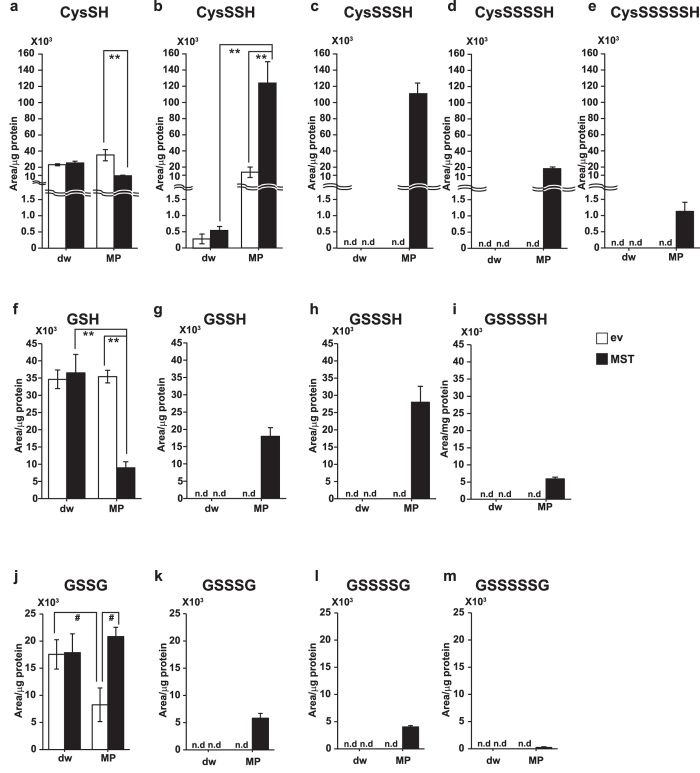
Cys-SS_n_H, GSS_n_H, and GSS_n_G production in lysates of COS cells expressing 3MST in the presence of 3MP. (**a**–**e**) Levels of cysteine (**a**) and production of Cys-SSH (**b**), Cys-SSSH (**c**), Cys-SSSSH (**d**) and Cys-SSSSSH (**e**) in lysates of cells expressing 3MST in the presence of 100 μM 3MP. Note that the reaction mixture of lysates contained approximately 1 μM cysteine and 10 μM GSH. Monobromobimane adducts of Cys-SS_n_H were detected by LC-MS/MS. **(f**–**i)** Levels of GSH (**f**) and production of GSSH (**g**), GSSSH (**h**) and GSSSSH (**i**) in lysates of cells expressing 3MST in the presence of 100 μM 3MP. Monobromobimane adducts of GSS_n_H were detected by LC-MS/MS. (**j**–**m**) Levels of GSSG (**j**) and production of GSSSG (**k**), GSSSSG (**l**), and GSSSSSG (**m**) in lysates of cells expressing 3MST in the presence of 100 μM 3MP. GSS_n_G were detected by LC-MS/MS. **p < 0.01, *p < 0.05 (n = 3) by ANOVA. All data expressed as mean ± s.e.m. N. d.: Not detected.

It is possible that the decrease in the levels of cysteine and GSH is due to the production of Cys-SSH and GSSH by 3MST. We analyzed these persulfurated species in lysates of COS cells expressing 3MST using LC-MS/MS. The levels of cysteine and GSH were decreased in cells expressing 3MST in the presence of 3MP as observed in the analysis using HPLC (Figs 3a–c, 4a and f). Only GSSG levels were decreased in control cells in the presence of 3MP, probably due to reduction of GSSG to GSH by 3MP (Fig. 4j). Cys-SH, Cys-SSH, Cys-SSSH, Cys-SSSSH, Cys-SSSSSH, GSH, GSSH, GSSSH, GSSSSH, GSSG, GSSSG, GSSSSG, and GSSSSSG were produced in lysates of cells expressing 3MST in the presence of 3MP, while these persulfurated species were below detectable levels in control cells except for Cys-SSH (Fig. 4 and Supplementary Figs S2 and S3). These observations suggest that the consumption of cysteine and GSH in the presence of 3MP in cells expressing 3MST is due to the production of Cys-SSH, GSSH, GSSG and their polysulfide counterparts (Cys-SS_n_H, GSS_n_H, and GSS_n_G, n ≥ 2).

The ratio of Cys-SS_n_H species production was examined in the presence of 3MP. The level of Cys-SSH was the greatest, followed by Cys-SSSH with almost the same level (Fig. 4b,c, and Supplementary Fig. S3a). Cys-SSSSH was much less than the former two and Cys-SSSSSH still less. It is interesting to note that Cys-SH was greatly consumed in the presence of 3MP, presumably in the production of Cys-SSH and Cys-SSSH in lysates which contain approximately 1 μM cysteine and 10 μM GSH (Fig. 4a–e, and Supplementary Fig. S3a).

The ratio of GSS_n_H species production was also examined. The level of GSSSH was greater than GSSH, but GSSSSH was much less (Fig. 4f–i, and Supplementary Fig. S3b). GSH is highly consumed in the presence of 3MP to produce GSSSH and GSSH (Fig. 4f–i, and Supplementary Fig. S3b). In the presence of 1 μM cysteine and 10 μM GSH that are approximately 1/100 of the physiological concentrations, Cys-SSSH and GSSSH were also produced to a similar level to Cys-SSH and GSSH.

GSSG was greater than GSSSG and GSSSSG (Fig. 4j–m, and Supplementary Fig. S3c). The oxidized forms of cysteine such as Cys-S-S-Cys were below detectable levels.

H_2_S_2_ and H_2_S_3_ as well as H_2_S were produced in the presence of 3MP in lysates of COS cells expressing 3MST as previously reported (Supplementary Figs S2 and S4a–c)^14^. Thiosulfate was also produced in a similar manner (Supplementary Figs S2 and S4d).

### Production of H_2_S_n_, Cys-SS_n_H and GSS_n_H by 3MST and its mutants

**Figure 5:**
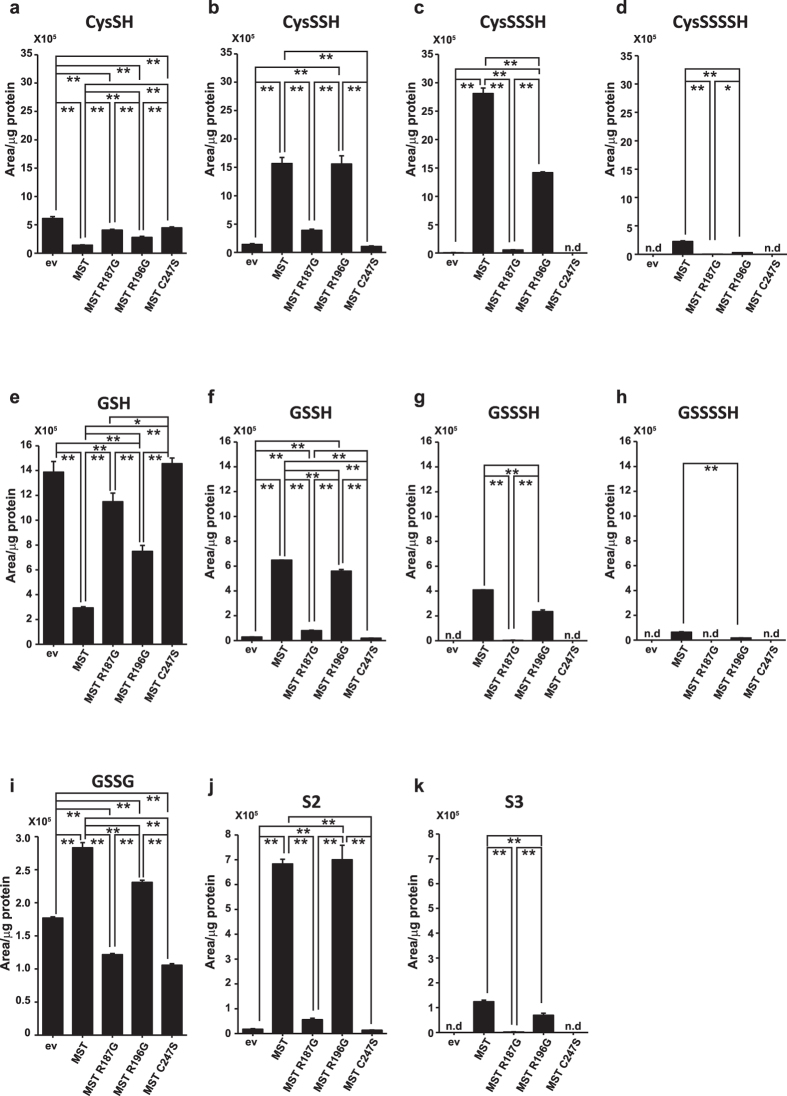
Cys-SS_n_H, GSS_n_H, GSSG, H_2_S_n_ production in lysates of COS cells expressing 3MST and its mutants in the presence of 3MP. (**a**–**d**) Levels of cysteine (**a**) and production of Cys-SSH (**b**), Cys-SSSH (**c**), and Cys-SSSSH (**d**) in lysates of cells expressing 3MST and its mutants in the presence of 100 μM 3MP. Note that the reaction mixture of lysates contained approximately 1 μM cysteine and 10 μM GSH. (**e**–**h**) Levels of GSH (**e**) and production of GSSH (**f**), GSSSH (**g**), GSSSSH (**h**) and GSSG (**i**) in lysates of cells expressing 3MST and its mutants in the presence of 100 μM 3MP. (**j**,**k**) Levels of H_2_S_2_ (**j**) and H_2_S_3_ (**k**) produced in lysates of cells expressing 3MST and its mutants in the presence of 100 μM 3MP. **p < 0.01, *p < 0.05 (n = 3) by ANOVA. All data expressed as mean ± s.e.m. N. d.: Not detected.

We examined the ability of 3MST and its mutants, R187G, R196G, and C247S to the differential production of H_2_S_n_, Cys-SS_n_H, and GSS_n_H from 3MP using COS cell lysates with LC-MS/MS. The levels of cysteine and GSH were decreased in cells expressing 3MST and R196G as observed previously (Figs 3a–c, 4a,f, 5a and e). Although 3MST and R196G produced almost the same levels of Cys-SSH and GSSH, 3MST generated greater amounts of Cys-SSSH, Cys-SSSSH, GSSSH and GSSSSH than R196G (Fig. 5a–h). Note that Cys-SSSH was produced more greatly than Cys-SSH in the presence of 1 μM cysteine and 10 μM GSH, approximately 1/100 of the physiological concentrations (Fig. 5b and c).

A similar result was obtained for the production of H_2_S_2_ and H_2_S_3_. Although 3MST and R196G produced H_2_S_2_ with a similar amount, 3MST generated H_2_S_3_ more greatly than R196G (Fig. 5j and k). 3MST produces reactive H_2_S_3_ more efficiently than R196G, and it may result in the effective production of CysSS_n_H and GSS_n_H (n ≥ 2) (Fig. 5k). The levels of GSSG were increased in 3MST and R196G to less extent (Fig. 5i).

The production of H_2_S_n_, Cys-SS_n_H and GSS_n_H was greatly decreased in C247S and R187G as predicted from the result that cysteine and GSH were not consumed by both mutants (Fig. 5a–h).

### Production of Cys-SSH and GSSH in whole cells

**Figure 6:**
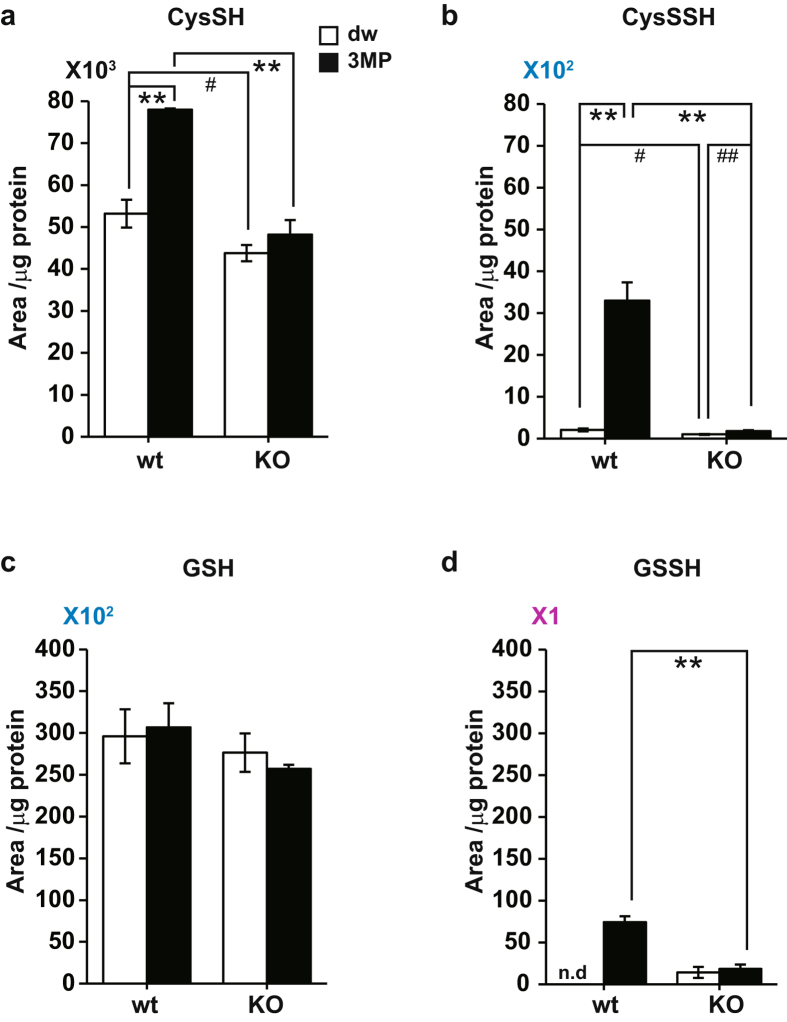
Production of Cys-SSH and GSSH in whole cells. (**a**,**c**) The levels of cysteine (**a**) and GSH (**c**) in the brain cell suspension, which were prepared from wild-type (wt) and 3MST-KO (KO) mice, exposed to 500 μM 3MP (filled bar) or to medium without 3MP (open bar). Note that approximately 10% of 3MP was incorporated into cells and metabolized by 3MST. Monobromobimane adducts of cysteine and GSH were detected by LC-MS/MS. (**b**,**d**) The production of Cys-SSH (**b**) and GSSH (**d**) in the brain cell suspension, which were prepared from wild-type (wt) and 3MST-KO (KO) mice exposed to 500 μM 3MP (filled bar) or to medium without 3MP (open bar). Monobromobimane adducts of Cys-SSH and GSSH were detected by LC-MS/MS. **p < 0.01, *p < 0.05 (n = 3 for wt, n = 5 for KO) by ANOVA, ^##^p < 0.01, ^#^p < 0.05 (n = 3 for wt, n = 5 for KO) by Student *t*-test. All data expressed as mean ± s.e.m.

The production of Cys-SSH and GSSH by whole cells was examined, using brain cell suspension prepared from wild type and 3MST-KO mice. We have previously shown that exogenously applied 3MP was incorporated into suspension cells and metabolized by 3MST to produce H_2_S_2_ and H_2_S_3_^14^. The level of Cys-SSH was greatly increased in cells prepared from wild-type mice, while no such increase was observed in cells prepared from 3MST-KO mice (Fig. 6b). A similar result was obtained for the production of GSSH (Fig. 6d). These observations confirmed that Cys-SSH and GSSH are produced by 3MST in brain cells. Other cysteine and GSH species with longer sulfur chains were below detectable levels.

H_2_S, H_2_S_2_, and H_2_S_3_ were produced in the brain of wild-type mice but not in 3MST-KO mice as previously reported (Supplementary Fig. S5a–c)^14^. Thiosulfate levels were also increased in the presence of 3MP in brain cells prepared from the wild-type mice compared to those from 3MST-KO mice (Supplementary Fig. S5d).

### Production of Cys-SS_n_H and GSS_n_H depends on concentrations of coexisting cysteine and GSH

Brain cells produce Cys-SSH and GSSH but not their polysulfide counterparts Cys-SS_n_H and GSS_n_H (Fig. 6). It is possible that the production of these persulfurated species depends on the existing concentrations of cysteine and GSH. To examine this possibility, the production of Cys-SS_n_H and GSS_n_H by 3MST in the presence of 3MP was investigated using recombinant 3MST in the presence of various concentrations of cysteine and GSH.

**Figure 7:**
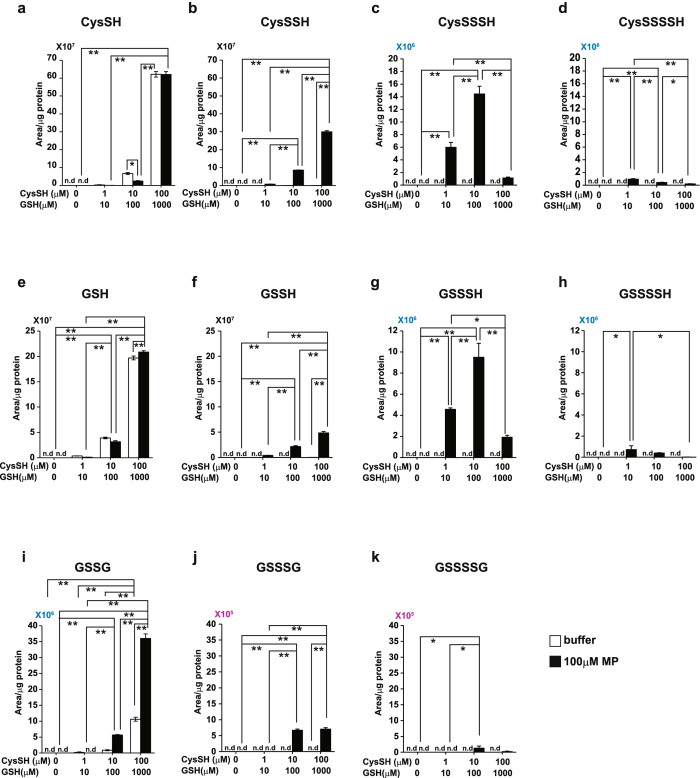
Cys-SS_n_H, GSS_n_H, and GSS_n_G production by recombinant 3MST in the presence of various concentrations of cysteine and GSH. (**a**–**d**) Levels of cysteine (**a**), and production of Cys-SSH (**b**), Cys-SSSH (**c**), and Cys-SSSSH (**d**) by recombinant 3MST in the presence of the indicated concentrations of cysteine and GSH with (filled bar) or without (open bar) 100 μM 3MP. Monobromobimane adducts of Cys-SS_n_H were detected by LC-MS/MS. (**e**–**h**) Levels of GSH (**e**), and production of GSSH (**f**), GSSSH (**g**), and GSSSSH (**h**) by recombinant 3MST in the presence of the indicated concentrations of cysteine and GSH with (filled bar) or without (open bar) 100 μM 3MP. Monobromobimane adducts of GSS_n_H were detected by LC-MS/MS. (**i**–**k**) Levels of GSSG (**i**), GSSSG (**j**), and GSSSSG (**k**) by recombinant 3MST in the presence of the indicated concentrations of cysteine and GSH with (filled bar) or without (open bar) 100 μM 3MP. GSS_n_G were detected by LC-MS/MS. **p < 0.01, *p < 0.05 (n = 3) by ANOVA. All data expressed as mean ± s.e.m. N. d.: Not detected.

Cys-SSH was maximally produced under a physiological condition in the presence of 100 μM cysteine with 1 mM GSH (Fig. 7b). Cys-SSSH and Cys-SSSSH were maximally produced in the presence of 10 μM cysteine with 100 μM GSH and 1 μM cysteine with 10 μM GSH, respectively (Fig. 7c and d). A similar result was obtained for the production of GSSH, GSSSH and GSSSSH (Fig. 7f–h). These observations suggest that Cys-SSH and GSSH are produced under physiological conditions, while Cys-SS_n_H and GSS_n_H are unstable under such conditions.

GSSG was maximally produced under a physiological condition in the presence of 100 μM cysteine with 1 mM GSH (Fig. 7i). Although the production of GSSSG was much less than that of GSSG, GSSSG production was greatest in the presence of 10 μM cysteine with 100 μM GSH as well as 100 μM cysteine with 1 mM GSH (Fig. 7j). The maximal production of GSSSSG was observed in the presence of 10 μM cysteine with 100 μM GSH (Fig. 7k). Cys-SS_n_-Cys were under detectable levels.

H_2_S was maximally produced under a physiological condition in the presence of 100 μM cysteine with 1 mM GSH (Supplementary Fig. S6a). The optimal production of H_2_S_2_ was achieved in the presence of 10 μM cysteine with 100 μM GSH, and that of H_2_S_3_ in the presence of 1 μM cysteine with 10 μM GSH or in the absence of cysteine and GSH (Supplementary Fig. S6b and c). Note that H_2_S_2_ as well as H_2_S more stably exists under physiological concentration of 100 μM cysteine with 1 mM GSH than H_2_S_3_ (Supplementary Fig. 6b and c). In contrast, H_2_S_3_ is rather unstable in the presence of physiological concentrations of cysteine and GSH, and their sulfane sulfur may be immediately transferred to cysteine and GSH to produce Cys-SSH and GSSH as well as onto cysteine residues of proteins to produce persulfurated proteins as shown in Fig. 2f and g. Thiosulfate was stably produced irrespective of the concentrations of cysteine and GSH (Supplementary Fig. S6d).

## Discussion

The present study showed that 3MST produces persulfurated species such as Cys-SSH and GSSH together with H_2_S_2_ and H_2_S_3_ as signaling molecules, and increases the levels of persulfurated proteins in a similar manner (Figs 1, 3–7, and Supplementary Figs S4 and S5)^14, 26, 40^.

H_2_S_3_ is more reactive than H_2_S_2_ to cysteine and GSH to produce CysSS_n_H and GSS_n_H. H_2_S_3_ was detected only 15 sec after the application of Na_2_S_3_, and most of them were transformed to H_2_S_2_ (Fig. 2). H_2_S_3_ must immediately be consumed to produce CysSS_n_H, GSS_n_H and sulfurated cysteine residues of proteins, while H_2_S_2_ reacts slower than H_2_S_3_. Therefore, H_2_S_2_ stays in the medium longer than H_2_S_3_ (Fig. 2).

Brain cells produced Cys-SSH and GSSH but not their polysulfide counterparts Cys-SS_n_H and GSS_n_H (Fig. 6). Cys-SS_n_H and GSS_n_H were preferably produced in the presence of lower than physiological concentrations of cysteine and GSH (Fig. 7). They are unstable in the presence of physiological concentrations of cysteine and GSH. Under physiological conditions, Cys-SSH and GSSH are major products, and even though Cys-SS_n_H and GSS_n_H are produced, they may be immediately reduced by cysteine and GSH. A similar result was obtained for H_2_S_2_ and H_2_S_3_ as main products (Supplementary Fig. S4)^14^. In contrast, thiosulfate is stable (Supplementary Fig. S5d).

**Figure 8:**
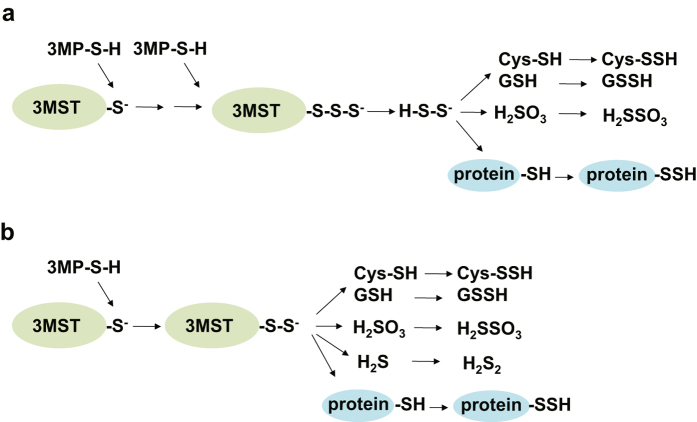
Potential mechanisms for the production of Cys-SSH, GSSH, persulfurated proteins and H_2_S_2_ by 3MST. (**a**) H_2_S_2_ produced by 3MST immediately reacts with cysteine, GSH, and cysteine residues to produce Cys-SSH, GSSH, and protein-SSH, respectively. (**b**) 3MST transfers sulfane sulfur to cysteine, GSH, and cysteine residues to produce Cys-SSH, GSSH, and protein-SSH, respectively.

There are two potential mechanisms for the production of these persulfurated species. One potential mechanism is that H_2_S_2_ and H_2_S_3_ produced by 3MST readily react with free cysteine and GSH to produce Cys-SSH and GSSH, and react with cysteine residues of proteins to generate persulfurated proteins (Figs 2 and 8a). Another potential mechanism is that 3MST transfers sulfur from 3MP to cysteine, GSH and H_2_S as well as to cysteine residues to produce Cys-SSH and GSSH, H_2_S_2_, and persulfurated proteins (Fig. 8b). It is also possible that the above two mechanisms proceed together to produce these persulfurated species.

Most cases with congenital 3MST deficiency are associated with mental retardation^46^. 3MST is localized to the synaptosomal fraction^26^, and H_2_S_n_ activate TRPA1 channels in astrocytes^21^, which surround the neuronal synapses and regulate the synaptic transmission by releasing a glio-transmitter D-serine^47^, leading to the facilitation of memory formation^40^. Anxiety-like behaviors were observed in 3MST knockout mice^48^. A concentration of neurotransmitter serotonin, of which the disturbance in the early development plays a critical role in the establishment of innate anxiety, was increased in the prefrontal cortex of 3MST knockout mice^48^. TRPA1 knockout mice also show anxiety-related behaviors^49^.

Parkin, a E3 ubiquitin ligase, whose loss of catalytic activity causes Parkinson’s disease^50^. Persulfuration of parkin enhances its catalytic activity, and persulfurated parkin is markedly depleted in brains of patients with Parkinson’s disease, suggesting that a loss of the activity of parkin may be pathologic^13^.

3MST is localized to cytosol and mitochondria^51^ and plays important roles in each compartment such as protection of cells from oxidative stress, transfer RNA (tRNA) synthesis^52–54^, and energy formation^55^. H_2_S_n_ sulfurate cysteine residues of Keap1 to release Nrf2 from Keap1/Nrf2 complex to the nucleus where Nrf2 upregulates the transcription of antioxidant genes, resulting in the increase in GSH production^23^. On the other hand, H_2_S produced in cytosol enhances the activity of cystine/glutamate antiporter to increase the transport of cystine, which is reduced to cysteine and used to produce GSH in cells^9^. H_2_S also enhances the activity of glutamate cysteine ligase (GCL), a rate limiting enzyme for GSH production^9^.

3MST supplies persulfide to thiolation of tRNA. Thio modification of uridine in the 2-position ensure accuracy of the genetic code and stabilization of tRNA structure. It was demonstrated that enzymes from rat brain as well as bacteria transfer sulfur from 3MP to thiolate tRNA^52, 53^. Recently, two isoforms of 3MST localized to cytosol and mitochondria were identified in human^54^. Cytosolic isoform thiolates cytosolic tRNA, while mitochondrial one has a dual localization in both mitochondria and the cystosol and not only functions as a direct supplier of persulfide to tRNA in the cytosol but also supplies sulfur for iron-sulfur cluster formation in mitochondria^54^.

Mitochondrial 3MST has also been proposed to be involved in the cellular bioenergetics^55^. H_2_S produced by 3MST may regulate mitochondrial electron transport and oxidative phosphorylation. Suppression of 3MST activity by siRNA decreased basal energetic parameters and prevented the stimulating effect of 3MP on mitochondrial bioenergetics^55^.

Cavallini *et al*. initially reported that CSE produces Cys-SSH from cystine, and CBS was recently proposed to have a similar activity to CSE^3, 4^. The controversial observations were interpreted to conclude that CSE and CBS do not produce Cys-SSH under physiological conditions^5^. Although CSE has high affinity to cystine with a K_m_ value of 30–70 μM^56^, the intracellular concentration of cystine in tissues is much lower than that required to advance the reaction. For example, 0.2 μM cystine in the liver is the greatest measured concentration among tissues, and is under the detectable level in the brain and heart^4^. Because CSE and CBS localize in the cytosol where cysteine and not cystine is the dominant form, it is difficult for both enzymes to produce Cys-SSH in the absence of enough cystine. CBS does not change bound sulfane sulfur levels in cell lysates nor in CBS-KO mice compared to their control (Fig. 1b)^26^. These observations support the notion that CBS does not produce Cys-SSH (Fig. 1b).

The present study shows the production of persulfurated species in cytosol. Other pathways to produce GSSH have been proposed in mitochondria and erythrocytes. Sulfur-quinone oxidoreductase (SQR) oxidizes H_2_S in mitochondria and utilizes GSH as a thiophilic acceptor to produce GSSH^57, 58^. In erythrocytes H_2_S binds to ferric-heme to be oxidized to form iron-bound polysulfides, which are reduced by GSH to produce GSSH^59^.

The present study provides a novel pathway to produce polysulfide species Cys-SSH, GSSH and persulfurated proteins as well as H_2_S_2_ and H_2_S_3_, and helps clarify the cellular signaling in which these persulfurated species are involved.

## Methods

### Animals

All experiments were approved and conformed to the guidelines set by the Small Animal Welfare Committee of the National Institute of Neuroscience, National Center of Neurology and Psychiatry. C57BL6 mice were purchased from Clea Japan Inc. (Tokyo, Japan), CBS-KO mice from Jackson Laboratory (Bar Harbor, USA), and 3MST-KO mice from Texas A&M Institute for Genomic Medicine (Texas, USA). Genotypes of CBS-KO and 3MST-KO were determined by polymerase chain reaction as previously reported^14, 41, 60, 61^.

### Measurement of bound sulfane sulfur

A whole brain homogenates were prepared with 9 volumes of ice-cold buffer consisting of 10 mM potassium phosphate (pH 7.4), 1% TritonX-100, 10 mM hydroxylamine, which was used to suppress the activity of PLP-dependent enzymes involved in enzymatic H_2_S production, and protease inhibitor cocktail “complete” (Roche Diagnostics, Mannheim, Germany) using a Potter type glass homogenizer with a Teflon pestle (1,500 rpm, 10 strokes). To lyse brain cell membranes, homogenates were mixed with vortex for 1 min on ice three times with 10 min intervals. The lysates were centrifuged at 12,000 × g for 10 min, and the supernatants were recovered.

For measurement of H_2_S released from bound sulfane sulfur, the method previously reported was used^26^. Briefly, 0.1 ml of supernatants (2.5 mg protein/ml) mixed with 0.1 ml of 15 mM DTT in 100 mM Tris/HCl (pH 9.0), was placed in a 15 ml centrifugation tube, then sealed and incubated at 37 °C for 50 min. After adding 0.4 ml of 1 M sodium citrate buffer, pH 6.0, the mixtures were incubated at 37 °C for 10 min with shaking at 125 rpm on a rotary shaker NR-3 (TAITEC) to facilitate release of bound sulfur as H_2_S gas from the aqueous phase. Two ml of approximate 14.5 ml of head-space gas was applied to a gas chromatograph (GC-14B; Shimazu, Kyoto, Japan) equipped with a flame photometric detector and a data processor C-R8A Chromatopac (Shimazu). A reaction mixture without samples was used as a control for a release of H_2_S from DTT.

### Recombinant 3MST

For recombinant 3MST: A previously reported method was used^14^. Briefly, 3-MST was prepared from fusions with glutathione S-transferase (GST) by the modified method previously reported by Smith and Johnson^62^. cDNA constructs of GST fusion proteins were incorporated in pGEX-6p-2 plasmid (GE Healthcare Life Sciences, Little Chalfont, USA) and transformed a bacterial line BL21. Bacteria were cultured in 400 ml M9 medium (6 g Na_2_HPO_4_, 3 g KH_2_PO_4_, 0.5 g NaCl, 1 g NH_4_Cl, 1 ml of 1 M MgSO_4_, 5.6 ml of 2 M glucose, 1 ml of 1% thiamine, 0.1 ml of 1 M CaCl_2_, and 100 μg/ml ampicillin in 1 l distilled water) at 20 °C for 24 hr in a shaker (Takasaki Scientific Instruments Corp. Saitama, Japan). When OD600 was increased to 0.6~0.8, isopropyl β–D-1-thiogalactopyranoside (IPTG) (Sigma, St. Louis, Missouri, USA) was added to make a final concentration of 0.1 mM and further cultured for 24 hr at 20 °C. Bacteria were collected by a centrifugation at 1,673 × g for 15 min and stored at −80 °C. Bacteria collected from 100 ml culture were lysed in 1 ml lysis buffer consisting of 858 μl PBS, 40 μl 25 × complete protease inhibitor cocktail (Hoffmann-La Roche, Basel, Switzerland) 1 μl 1 M DTT, 50 μl 10 mg/ml lysozyme, 1 μl 1 × 10^4^ U/ml DNA ase I, and 50 μl 20% Triton X on ice for 30~60 min, and then subjected to sonication. Lysates were centrifuged at 7,000 × g for 10 min by MX-100 (Tomy Seiko, Tokyo, Japan), and the supernatant was applied to GST Spin Trap column (GE Healthcare Life Sciences) and kept it for 10 min at room temperature. The spin column was centrifuged at 735 × g for 1 min and washed twice with 200 μl PBS. A hundred μl PreScission protease (GE Healthcare Life Sciences) solution containing 50 mM Tris (pH 8.0), 100 mM NaCl, 1 mM EDTA, 1 mM DTT was added to the column and incubated for 12~16 hr at 4 °C, and then 3MST, which had been excised from GST-fusion, was recovered by centrifugation at 735 × g for 1 min at room temperature. DTT was removed by PD spintrap G-25 (GE Healthcare Life Sciences).

### Cell lysates

The activity of enzymes expressed in COS-7 (COS) cells was examined as previously reported^14^. Briefly, COS cells were transfected with an expression plasmid encoding 3MST- or rhodanese-cDNA using TransIT-LT1 Transfection Reagent (Mirus Bio, Madison, WI, USA) following the procedure recommended by the manufacturer. After washed twice with PBS in the plates, cells were removed from the plate by scraping twice with each 0.3 ml BHM solution consisting of 0.32 M sucrose, 1 mM EDTA, 10 mM Tris-Cl (pH 7.0) and the complete protease inhibitor cocktail (Roche Applied Science, Upper Bavaria, Germany). The resultant 0.6 ml BHM solution containing cells was sonicated and centrifuged at 1,000 × g for 10 min, and the supernatant was used for measuring the enzyme activity. Fifty μl supernatant was mixed with 40 μl 100 mM KHPO_4_ (pH 7.0) and incubated for 5 min at 37 °C, and then 10 μl substrates (final 100 μM) such as 3-mercaptopyruvate (3MP, Sigma-Aldrich), Na_2_S (Wako Pure Cheimcal Industries, Osaka, Japan) or a control H_2_O were added to incubate at 37 °C for 15 min. The resultant reaction mixture was subjected to derivatization with monobromobimane (Life Technologies). The mixture was incubated in the presence of 2 mM monobromobimane for 20 min at room temperature, and then acetic acid was added to the final concentration of 1% and incubated 15 min on ice. The resulting reaction mixture was centrifuged at 15,000 × g for 10 min, and the supernatant was analyzed by LC-FL (Waters, Milford, MA, USA) and LC-MS/MS (Shimazu, Kyoto, Japan).

### Chemical interaction of Na_2_S_n_ with cysteine and GSH

One μl 10 mM cysteine (final conc. 100 μM) and 1 μl 100 mM GSH (final conc. 1 mM) were added to 97 μl medium produced by the mixture of 40 μl 100 mM KHPO_4_ (pH 7.0), 50 μl BHM and 7 μl distilled water, and mixed. One μl 1 mM Na_2_S_2_ or Na_2_S_3_ (final conc. 10 μM) was added to the medium and mixed. Ten μl each was taken from the mixture 0.25, 3, 10, 30 min after the application of Na_2_S_2_ or Na_2_S_3_, and labeled with 1 mM monobromobimane for 20 min at room temperature, and then acetic acid was added to the final concentration of 1% and incubated for 15 min on ice. The resulting reaction mixture was centrifuged at 15,000 × g for 10 min, and the supernatant was analyzed by LC-MS/MS. Na_2_S_2_, and Na_2_S_3_ for standard were obtained from Dojindo (Kumamoto, Japan).

### Suspensions of brain cells

The suspensions of brain cells was prepared by the modified method reported by Dutton *et al*.^63^. Briefly, brains of 3MST knockout mice or the wild-type mice were removed at the postnatal day 1 or 2 and submerged in the ice-cold Leiboritz’s L-15 medium (Life Technologies, Waltham Massachusetts, USA). After meninges were removed, brains were chopped to approximately 1 mm cubes with scissors in the medium. The suspended brain cubes were centrifuged at 100 × g, 4 °C for 20 sec to remove medium, and washed once with the medium. The brain cubes were incubated in 10 ml basic medium (3 mg/ml BSA fraction V (Sigma-Aldrich, St. Louis, MO, USA), 14 mM glucose (Sigma), 1.2 mM MgSO_4_ in Ca^2+^ free HBSS (Life Technologies) containing 0.025% trypsin EDTA (Life Technologies) for 15 min at 37 °C, and then 10 ml basic medium containing 6.4 μg/ml DNAse I (Sigma-Aldrich), 0.04 mg/ml Soy Bean Tripsin Inhibitor (SBTI) (Sigma) in HBSS was added and gently mixed. The supernatant was removed after centrifugation at 100 × g for 1 min at room temperature. Two ml basic medium containing 40 μg/ml DNAse I, 0.25 mg/ml SBTI, and 3 mM MgSO_4_ in HBSS was added to the brain cubes and mixed gently up and down with a pipette without making foams for 30 times. After a centrifugation at 100 × g for 1 min, cells were recovered and washed with 2 ml HBSS with Ca^2+^ and Mg^2+^ medium (Wako Pure Chemical Industries) containing 14 mM glucose (Sigma-Aldrich) for 3 times, and then preincubated at 37 °C for 1 hr in a shaker at 100 rpm (Taitec Bio-shaker BR-40LF, Saitama, Japan) before used for experiments.

### Production of Cys-SSH, GSSH, and H_2_S_n_ in whole cells

After preincubation for 1 hr at 37 °C, 300 μl suspensions of brain cells were incubated for 15 min at 37 °C in the presence of 500 μM 3MP (Sigma-Aldrich). After the exposure to 3MP or Na_2_S the suspensions of brain cells were centrifuged at 100 × g for 30 sec, and the supernatant was removed. Cells were suspended in 300 μl basic medium containing 14 mM glucose in HBSS with Ca^2+^ and Mg^2+^, and removed the supernatant after centrifugation at 100 × g for 30 sec. This step was repeated three times to wash out 3MP. Cells were sonicated in BHM solution and centrifuged at 15,000 × g for 10 min at room temperature. The supernatant was incubated in the presence of 2 mM monobromobimane for 20 min at room temperature, and then acetic acid was added to the final concentration of 1% and incubated for 15 min on ice. The resulting reaction mixture was centrifuged at 15,000 × g for 10 min, and the supernatant was analyzed by LC-FL and LC-MS/MS. Na_2_S_2_, and Na_2_S_3_ for standard were obtained from Dojindo (Kumamoto, Japan).

### LC-FL analysis

Samples derivatized with monobromobimane (mBB) (Life Technologies) were separated with a Waters Symmetry C18 (ID, 250 × 4.6 mm) column (Waters Corp., Milford, MA, USA) with mobile phase A(0.25% acetic acid in H_2_O, pH 3.9), B(0.25% acetic acid: methanol = 7:3) and C(0.25% acetic acid: methanol = 1:1) with a linear gradient from A:B = 65:35 to 2:8 in 8 min with a flow rate of 1.5 ml/min, and remained with 100% B for additional 10 min, and then changed to 100% C in the following 6 min with a flow rate of 1.0 ml/min. The monobromobimane adduct was monitored with a scanning fluorescence detector (Waters 2475) with an excitation wavelength of 370 nm and an emission wavelength of 485 nm.

### LC-MS/MS analysis

Samples derivatized with monobromobimane (mBB) (Life Technologies) were analyzed by the triple-quadrupole mass spectrometer coupled to HPLC (Shimadzu LCMS-8040). Samples were subjected to a reverse phase Symmetry C18 HPLC column (4.6 × 250 mm, Waters) at the flow rate of 1.0 ml/min. The mobile phase consisted of (A) 0.1% formic acid in water and (B) 0.1% formic in methanol. Samples were separated by eluting with a gradient: 5% B at 0–5 min and 5–90% B at 5–25 min. The column oven was maintained at 40 °C. The effluent was subjected to the mass spectrometer using an electrospray ionization (ESI) interface operating in the positive-ion mode. The source temperature was set at 400 °C, and the ion spray voltage was at 4.5 kV. Nitrogen was used as a nebulizer and drying gas. The tandem mass spectrometer was tuned in the multiple reaction monitoring mode to monitor mass transitions in positive ion mode: CysS-mBB *m/z* 312 → 192, CysSS-mBB *m/z* 344 → 192, CysSSS-mBB *m/z* 376 → 192, CysSSSS-mBB *m/z* 408 → 192, GS-mBB *m/z* 498 → 225, GSS-mBB *m/z* 530 → 192, GSSS-mBB *m/z* 562 → 192, GSSSS-mBB *m/z* 594 → 192, GSSG *m/z* 613 → 355, GSSSG *m/z* 645 → 387, GSSSSG *m/z* 677 → 339, GSSSSSG *m/z* 709 → 371, mBB-S-mBB *m/z* 432.45 → 192, mBB-S_2_-mBB *m/z* 464.55 → 192, mBB-S_3_-mBB *m/z* 496.60 → 192, HS_2_O_3_-mBB *m/z* 305 → 225.

### Statistical analysis

All the statistical analyses of the data were performed using Microsoft Excel 2010 for Window 7 (Microsoft, Redmond, WA, USA) with the add-in software Statcel2 (OMS, Saitama, Japan). Differences between 2 groups were analyzed with Student’s *t* test. The differences between 3 or more groups were analyzed with one-way analysis of variance (ANOVA). Post hoc multiple comparisons were made using the Tukey-Kramer test.

## Electronic supplementary material

Supplementary Information
