# Supplementary material for: 3-Mercaptopyruvate sulfurtransferase produces potential redox regulators cysteine- and glutathione-persulfide (Cys-SSH and GSSH) together with signaling molecules H2S2, H2S3 and H2S
Source: Sci Rep. 2017 Sep 5;7:10459. doi: 10.1038/s41598-017-11004-7 (PMC5585270; doi:10.1038/s41598-017-11004-7)
Supplement: Supplementary file 1 — Supplementary Information [file 41598_2017_11004_MOESM1_ESM.pdf]

Supplementary Information

3-Mercaptopyruvate sulfurtransferase produces potential redox regulators cysteine- and glutathione-persulfide (Cys-SSH and GSSH) together with signaling molecules H_2S_2, H_2S_3 and H_2S

Yuka Kimura [1], Shin Koike [2], Norihiro Shibuya [1], David Lefer [3], Yuki Ogasawara [2], Hideo Kimura [1 #].

1. National Institute of Neuroscience, National Center of Neurology and Psychiatry, 4-1-1 Ogawahigashi, Kodaira, Tokyo 187-8502, Japan.
2. Department of Analytical Biochemistry, Meiji Pharmaceutical University, 2-552-1 Noshio, Kiyose, Tokyo 204-8588, Japan.
3. Department of Pharmacology and Experimental Therapeutics and Cardiovascular Center of Excellence, LSU Health Science Center, New Orleans, LA 70112, USA.

# Correspondence should be addressed to Hideo Kimura, National Institute of Neuroscience, National Center of Neurology and Psychiatry, 4-1-1 Ogawahigashi, Kodaira, Tokyo 187-8502, Japan. E-mail: kimura@ncnp.go.jp

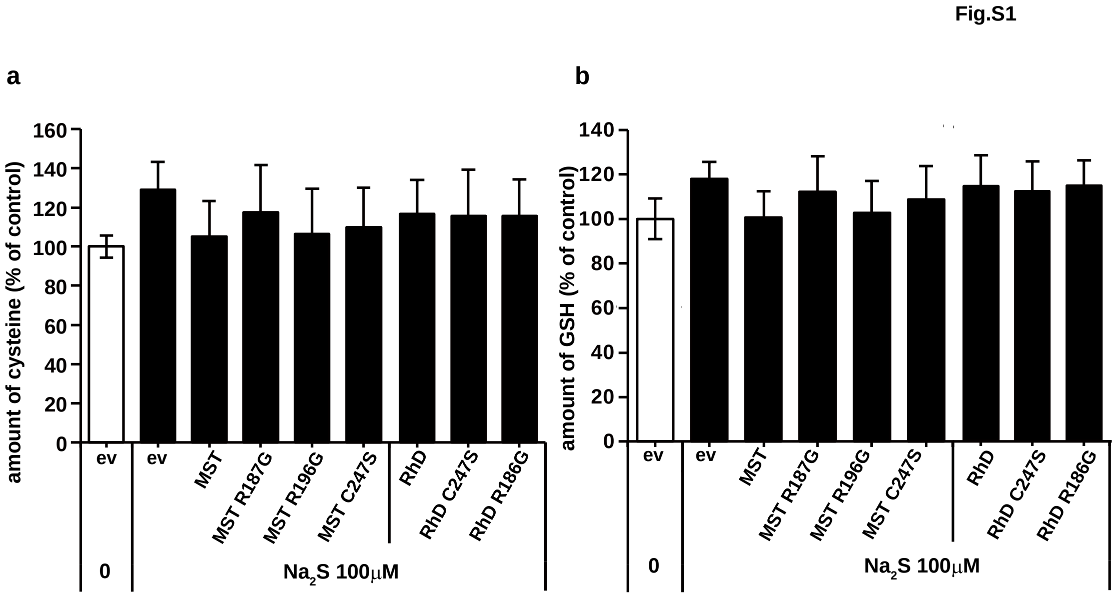

Supplementary Figure S1.

H_2S does not change cysteine and GSH levels. **a** and **b**. There is no significant difference in the levels of cysteine (**a**) and GSH (**b**) in cells expressing 3MST or rhodanese (RhD) and their mutants in the presence of 100 μM Na_2S. Monobromobimane adducts of cysteine and GSH were detected by LC with a fluorescence detector (n = 5). All data expressed as mean ± s.e.m.

Fig.S2

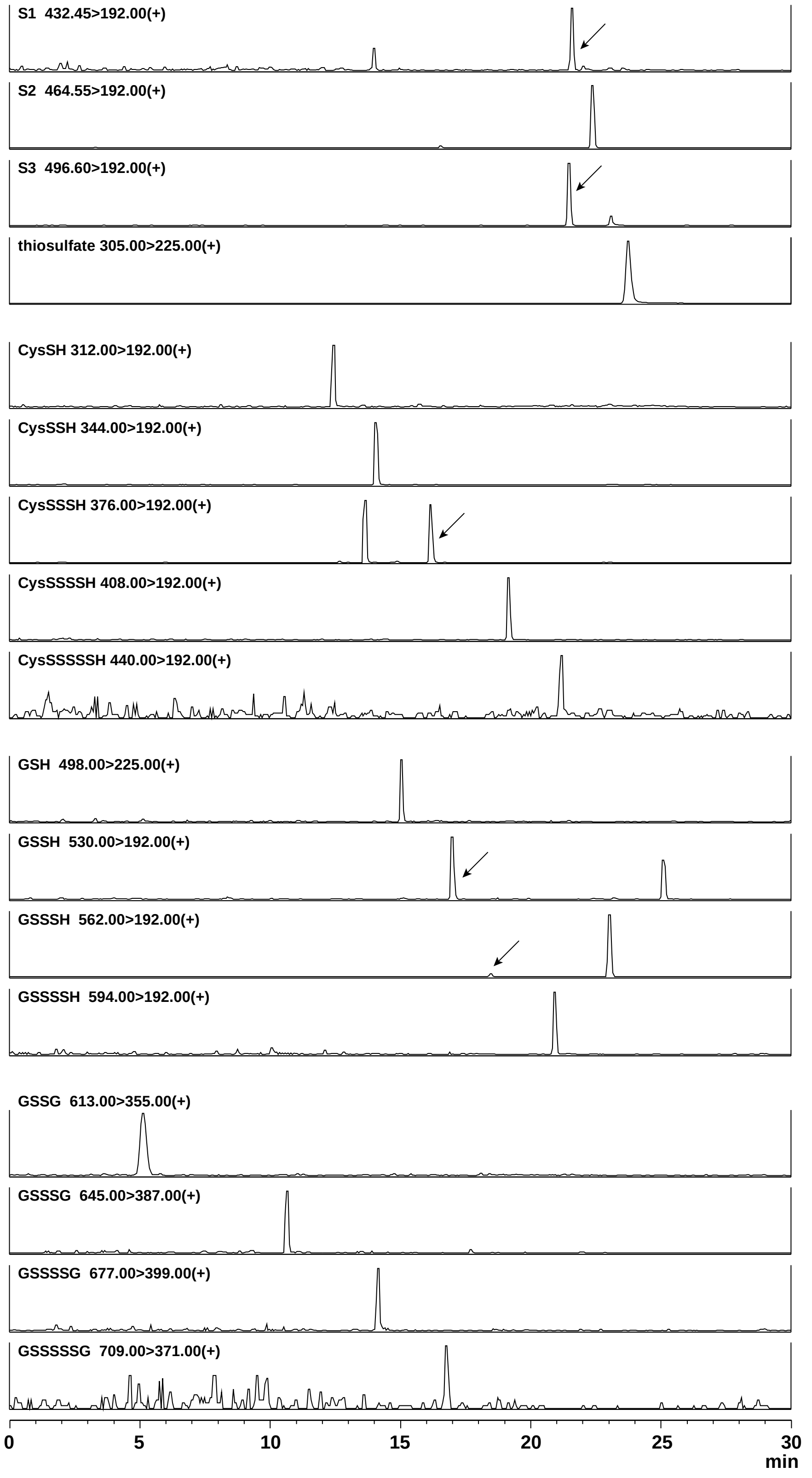
S1 432.45>192.00(+)
S2 464.55>192.00(+)
S3 496.60>192.00(+)
thiosulfate 305.00>225.00(+)
CysSH 312.00>192.00(+)
CysSSH 344.00>192.00(+)
CysSSSH 376.00>192.00(+)
CysSSSSH 408.00>192.00(+)
CysSSSSSH 440.00>192.00(+)
GSH 498.00>225.00(+)
GSSH 530.00>192.00(+)
GSSSH 562.00>192.00(+)
GSSSSH 594.00>192.00(+)
GSSG 613.00>355.00(+)
GSSSG 645.00>387.00(+)
GSSSSG 677.00>399.00(+)
GSSSSSG 709.00>371.00(+)
0
5
10
15
20
25
30
min

Supplementary Figure S2.

A representative chromatogram of LC-tandem mass spectrometry (LC-MS/MS). LC-MS/MS chromatograms are shown for monobromobimane adducts of S_1, S_2, S_3, thiosulfate, Cys-SH, Cys-SSH, Cys-SSSH, Cys-SSSSH, Cys-SSSSSH, GSH, GSSH, GSSSH, and GSSSSH. GSSG, GSSSG, GSSSSG, and GSSSSSG were also detected. Note that S_1, S_2 and S_3 are detected as di-bimane adducts, while others are mono-bimane adducts.

Fig.S3

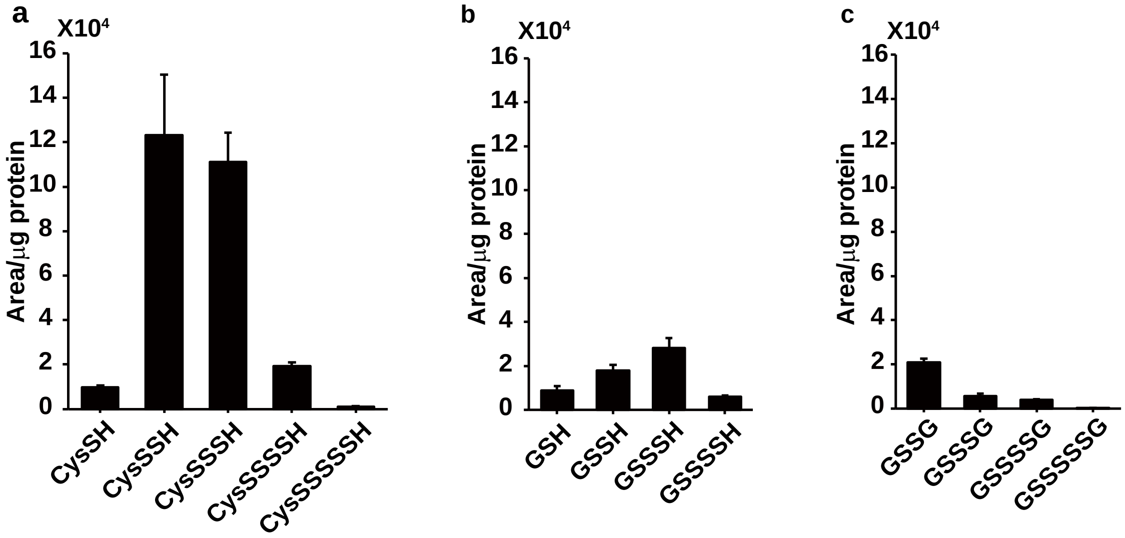

Supplementary Figure S3.

Comparison of the levels of Cys-SS_nH, GSS_nH and GSS_nG produced by 3MST. **a-c**. The relative levels of Cys-SS_nH (**a**), GSS_nH (**b**), and GSS_nG (**c**) produced in lysates of COS cells expressing 3MST in the presence of 100 μM 3MP (n = 3). Note that the reaction mixture of lysates contained approximately 1 μM cysteine and 10 μM GSH. Note that data were extracted from Fig. 3. All data expressed as mean ± s.e.m.

Fig.S4

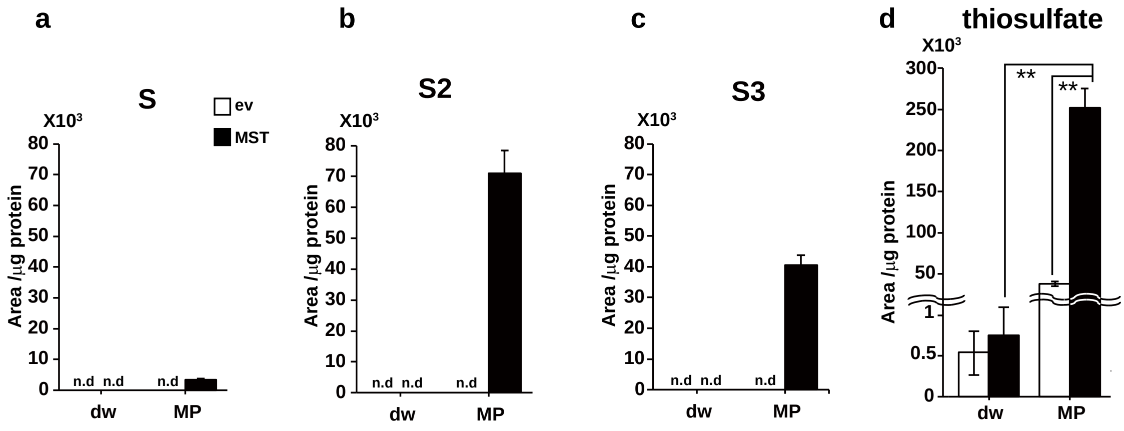

Supplementary Figure S4.

Production of H_2S, H_2S_2, H_2S_3 and thiosulfate in lysates of cells expressing 3MST.

a-d. H_2S (**a**), H_2S_2 (**b**), H_2S_3 (**c**) and thiosulfate (**d**) were produced in lysates of cells expressing 3MST (filled bar) in the presence of 100 μM 3MP, but they were much less in those transfected with an empty vector (open bar) (n = 3). Note that the reaction mixture of lysates contained approximately 1 μM cysteine and 10 μM GSH. ** $p < 0.01$ by ANOVA. All data expressed as mean $\pm$ s.e.m. N. d.: Not detected.

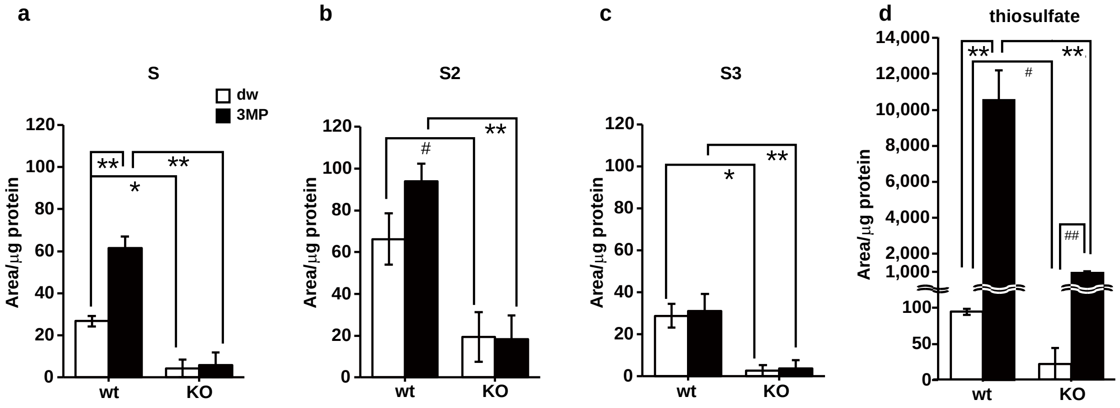

Supplementary Figure S5.

Production of H_2S, H_2S_2, H_2S_3 and thiosulfate in whole cells. **a-d**. Production of H_2S (**a**), H_2S_2 (**b**), H_2S_3 (**c**) and thiosulfate (**d**) in brain cell suspension prepared from the wild-type (wt) mice and 3MST-KO (KO) were shown after cells were exposed to 500 μM 3MP (filled bar) or medium without 3MP (open bar) (n = 3 for wt, n = 5 for KO). Note that approximately 10% of 3MP was incorporated into cells 15 min after exposure to 3MP and metabolized by 3MST [14]. ** $p < 0.01$, * $p < 0.05$ by ANOVA. # $p < 0.05$ by Student t-test. All data expressed as mean ± s.e.m.

Fig.S6

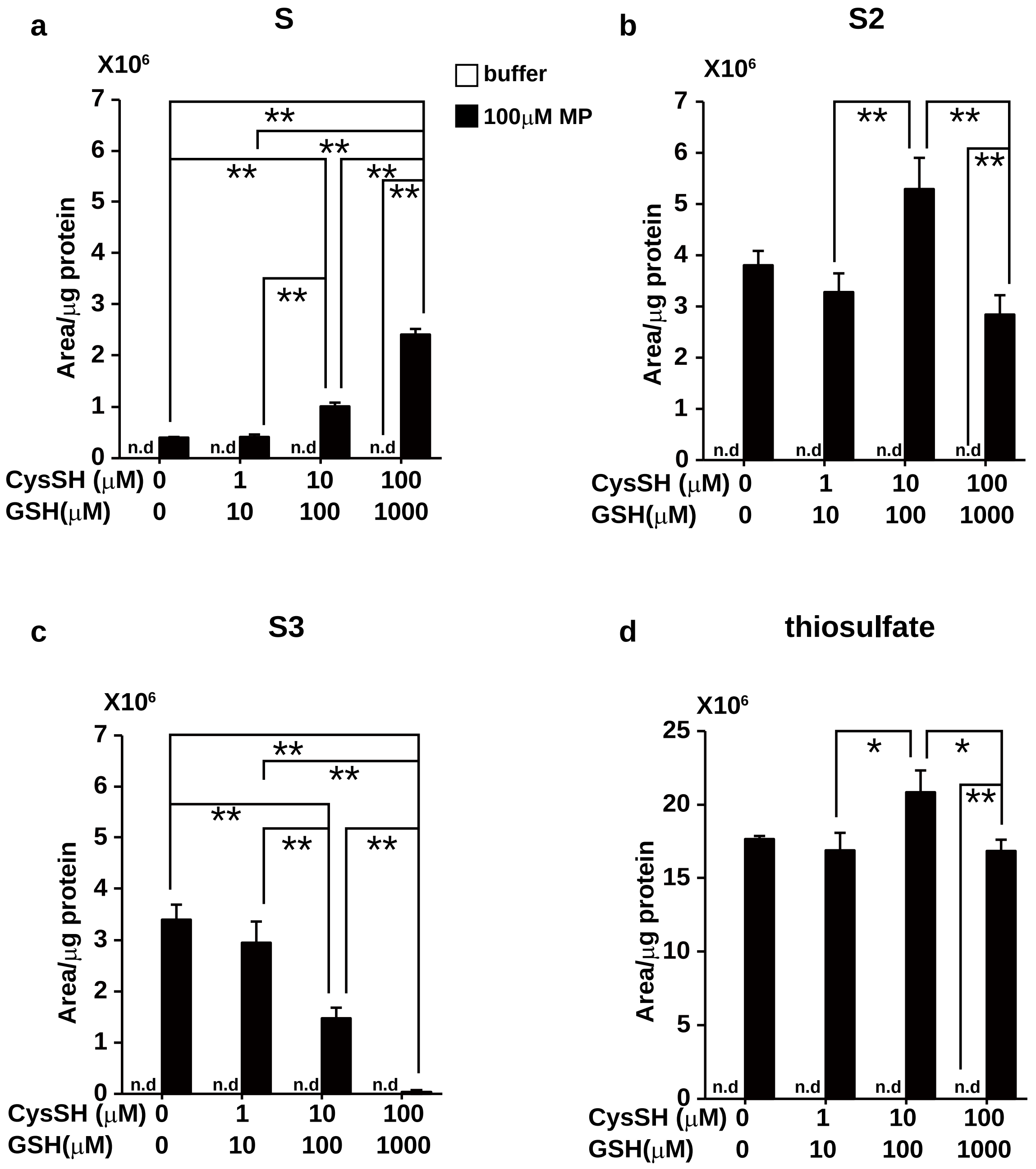

Supplementary Figure S6.

Production of H_2S, H_2S_2, H_2S_3 and thiosulfate by recombinant 3MST in the presence

of various concentrations of cysteine and GSH. **a**-**d**. The levels of H_2S (**a**), H_2S_2 (**b**), H_2S_3 (**c**) and thiosulfate (**d**) by recombinant 3MST with 100 μM 3MP in the presence of indicated concentrations of cysteine and GSH. (n = 3) ** $p < 0.01$, * $p < 0.05$ by ANOVA. All data expressed as mean ± s.e.m. N. d.: Not detected.
